# A Public Health Preparedness Logic Model: Assessing Preparedness for Cross-border Threats in the European Region

**DOI:** 10.1089/hs.2016.0126

**Published:** 2017-10-01

**Authors:** Michael A. Stoto, Christopher Nelson, Elena Savoia, Irina Ljungqvist, Massimo Ciotti

**Keywords:** Public health preparedness, Incident recognition, Risk characterization, Policy development and adaptation, Emergency risk communication, Crisis management

## Abstract

Improving preparedness in the European region requires a clear understanding of what European Union (EU) member states should be able to do, whether acting internally or in cooperation with each other or the EU and other multilateral organizations. We have developed a preparedness logic model that specifies the aims and objectives of public health preparedness, as well as the response capabilities and preparedness capacities needed to achieve them. The capabilities, which describe the ability to effectively *use* capacities to identify, characterize, and respond to emergencies, are organized into 5 categories. The first 3 categories—(1) assessment; (2) policy development, adaptation, and implementation; and (3) prevention and treatment services in the health sector—represent what the public health system must accomplish to respond effectively. The fourth and fifth categories represent a series of interrelated functions needed to ensure that the system fulfills its assessment, policy development, and prevention and treatment roles: (4) coordination and communication regards information sharing within the public health system, incident management, and leadership, and (5) emergency risk communication focuses on communication with the public. This model provides a framework for identifying *what* to measure in capacity inventories, exercises, critical incident analyses, and other approaches to assessing public health emergency preparedness, not *how* to measure them. Focusing on a common set of capacities and capabilities to measure allows for comparisons both over time and between member states, which can enhance learning and sharing results and help identify both strengths and areas for improvement of public health emergency preparedness in the EU.

## Abstract 2

Improving preparedness in Europe requires a clear understanding of what EU member states should be able to do, whether acting internally or in cooperation with each other. The authors developed a preparedness logic model that specifies the aims and objectives of public health preparedness, as well as the response capabilities and preparedness capacities needed to achieve them.

Effectively responding to emerging infectious disease threats requires both a strong national-level response and effective coordination across countries. In 2013, the European Union (EU) adopted Decision No. 1082/2013/EU, which seeks to strengthen public health emergency preparedness planning and response within and across EU member states. Decision 1082 (see Figure 1) focuses on “cross-border threats to health,” including biological, chemical, and environmental threats, and threats of unknown origin, or any event that may constitute a public health emergency of international concern under the International Health Regulations (2005).^1^

**Figure 1.**
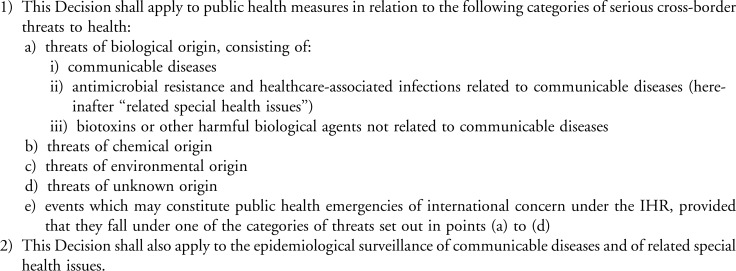
Scope of Decision No. 1082/2013/EU

Assessing—and improving—preparedness requires that member states possess a clear understanding of what actions they are able to take in response to a threat, whether acting internally, in cooperation with each other, or with EU and other multilateral organizations. However, measuring the basic components of preparedness is difficult, as serious public health emergencies are relatively infrequent and possess many aspects specific to their respective contexts, leaving few opportunities to assess outcomes by direct observation in critical incident reviews.^2^ Therefore, public health system researchers sometimes develop a logic model to address these challenges, identifying the factors that, based on scientific evidence and practitioner experience, contribute to positive outcomes.^3^ A preparedness logic model should specify the aims and objectives of public health preparedness, as well as the response capabilities and preparedness capacities needed to achieve them. Analyzing the response to actual incidents can strengthen the evidence base supporting a logic model. However, even without a strong evidence base, logic models can be an effective method for identifying provisional measures based on current knowledge.

In order to enable EU member states to identify preparedness gaps and to assist the European Centre for Disease Prevention and Control (ECDC) in developing a set of competency-based training programs, we adapted a preparedness logic model, originally created to capture elements of the US preparedness enterprise,^4,5^ using 4 approaches.

First, we examined the results of a literature review of 6 cross-border emergencies that occurred in Europe to understand differences in capabilities in the European context.^6^ The events were: biological hazards (an outbreak of *E. coli* in 2011 and pandemic influenza H1N1 in 2009 and 2010), chemical hazards (a sludge reservoir breach in 2010 in Hungary threatening the Danube River and melamine contamination of milk in 2008 in products imported from China), and hazards of environmental origin (a heat wave affecting several European countries in 2003 and a volcanic ash cloud originating in Iceland in 2010).

Second, we reviewed reports of peer review visits on the European response to Ebola in 2014 conducted by the ECDC in 3 EU member states. (Examples from the review of these incidents are mentioned below to illustrate the meaning of the capabilities, but due to space limitations, not all of the examples we used are listed.)

Third, we reviewed existing competency statements developed by ECDC for public health epidemiologists,^7^ microbiologists,^8^ and healthcare infection control experts.^9^ We also consulted the scientific literature about public health emergency preparedness and guidance.^10,11^

Finally, we invited participants at the 2015 annual meeting of the ECDC National Focal Points for Preparedness and Response to identify which capabilities were implemented during the responses to the recent Ebola and Middle Eastern respiratory syndrome (MERS) outbreaks.

The following section provides a series of definitions that anchored the development of the logic model, particularly in distinguishing between capabilities and capacities. We describe the method used for identifying both the critical capabilities and capacities in the European context.

## Defining Preparedness

Our logic model is grounded in the definition of public health emergency preparedness developed by Nelson and colleagues:

… the capability of the public health and health care systems, communities, and individuals, to prevent, protect against, quickly respond to, and recover from health emergencies, particularly those whose scale, timing, or unpredictability threatens to overwhelm routine capabilities. Preparedness involves a coordinated and continuous process of planning and implementation that relies on measuring performance and taking corrective action.^12^

This definition's core concept is the “public health emergency preparedness system,” a complex network of individuals and organizations that play critical roles in creating the conditions for health. The Institute of Medicine (IOM) represents this system with the government public health infrastructure at the core and the healthcare delivery system, civil protection agencies, employers and businesses, the media, academia, and other public and private community organizations as important components.^13^ While these actors operate as separate entities, a robust public health emergency preparedness system requires that all components work together when necessary.

Our logic model incorporates a fundamental distinction between capacities (eg, infrastructure, trained personnel, policies and procedures) and capabilities, or the ability to *use* these capacities to effectively identify, characterize, and respond to emergencies. For example, having strong laboratories and skilled microbiologists may be insufficient if they cannot be mobilized in a timely manner, or if laboratory results cannot be shared with and acted on by decision makers. Capacities and capabilities are both important for an effective emergency response; however, depending on the context, different kinds of capacities are needed to achieve the required capabilities. The logic model focuses measurement efforts largely on capabilities, allowing different EU member states to determine how to best achieve them in their own context. For instance, member states with widespread integrated national electronic medical record systems might rely on them for syndromic surveillance to detect disease outbreaks, but countries or regions with less well-developed systems might rely on more traditional surveillance methods.

Capacities represent the means that a country uses to achieve its preparedness capabilities but necessarily reflect variations in member states' government and private-sector organizations. In some member states, for instance, surveillance is conducted at the national level, whereas in others surveillance and other public health activities are conducted at the local level. Some countries have a national, government-run healthcare delivery system, whereas others have a regional system with significant private-sector involvement. Consequently, it is difficult to specify capacities in a way that applies to all EU member states. Capabilities, on the other hand, describe what member states are expected to achieve during an emergency and can be described in a consistent way for all countries. For this reason, this analysis focuses on capabilities rather than capacities.

The capabilities and capacities in this logic model represent a theoretical framework for identifying the key dimensions of preparedness member states need to assess. Capacity inventories, exercises, critical incident analyses, and other approaches are all useful methods for conducting the assessments, but they are beyond the scope of this article.^5,14^

## ECDC Logic Model

**Figure 2.**
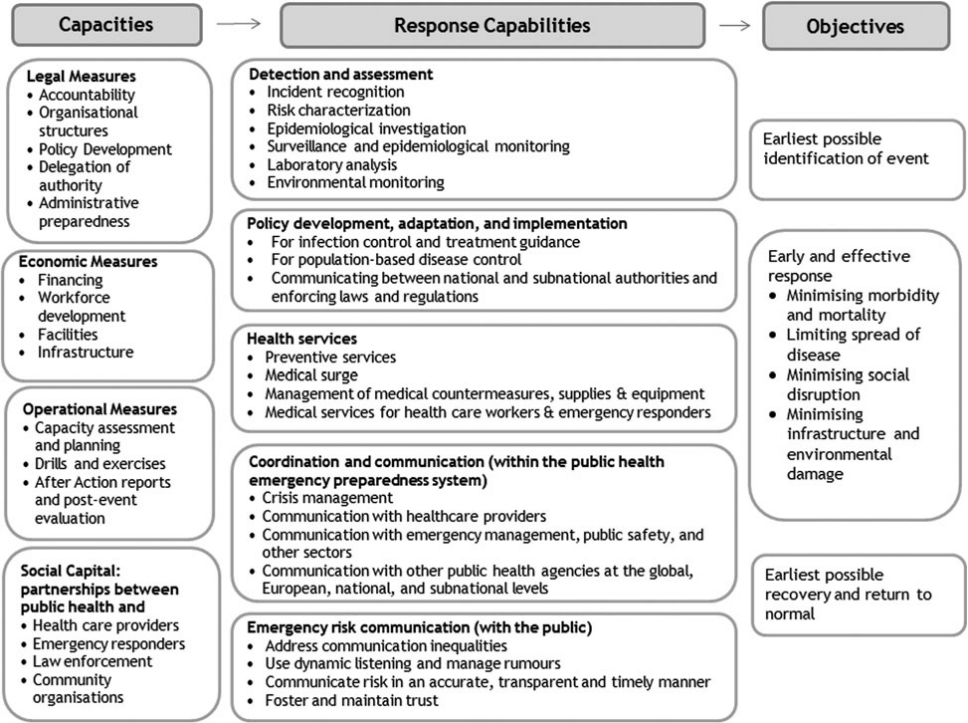
Logic Model for Public Health Preparedness in EU Member States

**Figure 3.**
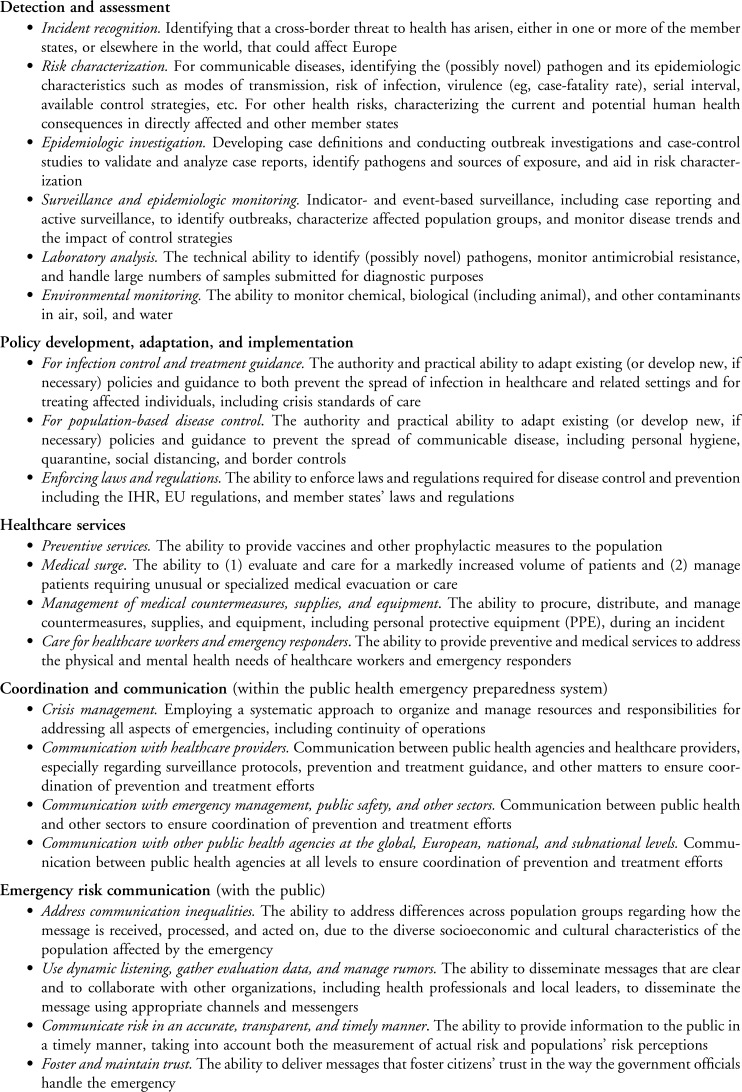
Proposed ECDC Public Health Preparedness Capabilities

Below we describe the logic model (Figure 2) that emerged from the process described above, beginning with descriptions of the capacities and capabilities included in the model. The capabilities are described in more detail in Figure 3. Consistent with Decision 1082, the logic model focuses on “cross-border threats to health.” However, most of the capacities and capabilities are also important for responding to public health emergencies that are entirely confined to member states.

### Capacities

The public health emergency preparedness logic model begins with Potter and colleagues' grouping of public health emergency preparedness capacities into legal, economic, and operational domains.^15^ Our research suggests the importance of adding “social capital,” which describes the partnerships and informal relationships between individuals and organizations critical to effective emergency operations and community resilience.^16,17^ The Toolkit for Assessing Health System Capacity for crisis management,^10^ created by WHO Europe, the WHO's Joint External Evaluation Tool,^11^ and the WHO's Strategic Framework For Emergency Preparedness^18^ also describe important public health emergency preparedness capacities.

### Capabilities

Given the number of capabilities in the logic model, it is helpful to organize them into high-level categories. The first 3 categories—(1) assessment; (2) policy development, adaptation, and implementation; and (3) health services—represent what the public health system must accomplish to respond effectively. The fourth and fifth categories represent a series of interrelated functions needed to ensure that the system fulfills its assessment, policy development, and prevention and treatment roles: (4) coordination and communication concerns information sharing within the public health system, incident management, and leadership, and (5) emergency risk communication focuses on communication with the public.

Evaluation and training are critical components of the preparedness cycle but are not “response capabilities” per se. We recognize that conducting post-incident reviews, and sometimes research, in a timely manner is of critical importance for system learning. This can require performing these analyses or collecting appropriate data during or shortly after an event. Similarly, an effective response can include just-in-time training and exercises. However, these activities are more properly categorized as capacity development, rather than response capabilities, despite their timing.

The case examples cited in the following discussion are intended to illustrate the concepts and demonstrate the importance of these capabilities for cross-border threats to health. They also document the process we used to develop the capabilities described above.

***1. Detection and Assessment***

As a whole, assessment capabilities enable member states' preparedness systems to recognize and characterize a threat, monitor its impact on the population, and evaluate the efficacy of interventions to contain the threat. Assessment depends on having laboratory and surveillance capacities, including appropriate legal arrangements, in place before an event. Information derived from assessment activities must be communicated to all segments of the public health preparedness system and the public in order to support policy development and implementation, prevention, and treatment efforts.

***Incident recognition.*** *Identifying that a cross-border threat to health has arisen, either in one or more of the member states or elsewhere in the world, that could affect Europe.* A notification to the Robert Koch Institute (RKI) of a small cluster of hemolytic uremic syndrome (HUS) cases in children in Germany prompted further investigation, leading to the recognition of a widespread *E. coli* outbreak. Similarly, the contamination of infant formula with melamine was recognized as a hazard across Europe when the International Food Safety Authorities Network (INFOSAN) emergency surveillance system published an alert.

***Risk characterization.*** *For communicable diseases, identifying the (possibly novel) pathogen and its epidemiologic characteristics, such as modes of transmission, risk of infection, virulence (eg, case-fatality rate), serial interval, available control strategies, and so on. For other health risks, characterizing the current and potential human health consequences in directly affected and other member states.* In Ebola, the challenge was characterizing the risks of transmission associated with repatriated healthcare workers and unknown imported cases. The ECDC's Rapid Risk Assessment process was designed to provide this information at the European level, but member states had to make their own assessments based on the context and conditions of their countries. For melamine, the European Food Safety Authority (EFSA) developed theoretical exposure scenarios of biscuits and chocolate containing milk powder for both adults and children to assess risk to individuals and populations. In the heat wave, the challenge was to identify the most vulnerable groups, such as the elderly.

***Epidemiologic investigation****. Developing case definitions and conducting outbreak investigations and case-control studies to validate and analyze case reports, identify pathogens and sources of exposure, and aid in risk characterization.* During the *E. coli* outbreak, an RKI research team interviewed cases and conducted case-control studies to investigate infection risks and identify the way infection was being transmitted. For the same incident, the ECDC published a standard case definition to coordinate investigations across multiple countries.

***Surveillance and epidemiologic monitoring****. Indicator- and event-based surveillance, including case reporting and active surveillance, to identify outbreaks, characterize affected population groups, and monitor disease trends and the impact of control strategies.* In the *E. coli* event, active surveillance was used to identify hemolytic uremic syndrome cases beyond those that triggered the initial epidemiologic investigation. During the Ebola outbreak, one challenge for some ECDC member states was identifying imported cases through border screening. During the sludge incident, those residing in contaminated villages had their health status carefully monitored.

***Laboratory analysis****. The technical ability to identify (possibly novel) pathogens, monitor antimicrobial resistance, and handle large numbers of samples submitted for diagnostic purposes.* This capability reflects a member state's ability to use existing laboratory capacity effectively during an incident in support of the other capabilities in the assessment section. This capability includes sequencing technologies and nonsequencing typing methodology, which can be helpful in confirming whether cases and events are linked.^8^ For example, in the Ebola outbreak, European mobile laboratories were used to support member states' efforts. In the *E. coli* outbreak, the EU reference laboratory for *E. coli* (EU-RL) and its associated networks were essential in characterizing the Shiga toxin–producing *E. coli* (STEC) strain. During the 2009 H1N1 pandemic, laboratory capacity for conducting a high volume of diagnostic tests was essential for effective response.

***Environmental monitoring****. The ability to monitor chemical, biological (including animal), and radiological contaminants in air, soil, and water.* Environmental monitoring also includes the laboratory capacity to analyze environmental samples. Effectively responding to the volcanic ash event required extensive geophysical monitoring of the Eyjafjallajökull volcano before, during, and after the eruption. Similarly, the Hungarian sludge incident required extensive postevent monitoring of water quality, fish, and other environmental aspects.

***2. Policy Development, Adaptation, and Implementation***

The group of policy development, adaptation, and implementation capabilities reflect member states' ability to adapt existing authorities and policies to the new and emerging circumstances of a cross-border threat to health, and to enforce both existing and new laws and regulations needed to implement these policies. Adaptation and implementation must be timely and flexible, reflecting developing information about the threat. These capabilities are focused on the national level, although it is recognized that a lack of consistency within member states, or among bordering member states, can erode public confidence. All of these activities require communication and coordination across a variety of actors working at the global, European, national, and subnational levels, and these supporting capabilities are described below. The capabilities in this section apply to the development and implementation of substantive policies, regulations, and official guidance regarding infection control and disease treatment in clinical settings and for population-based disease control activities.

***For infection control and treatment guidance****. The authority and practical ability to adapt existing (or develop new, if necessary) policies and guidance to both prevent the spread of infection in healthcare and related settings and for treating affected individuals, including crisis standards of care.* For example, during the Ebola outbreak, infection control practices for the use of personal protective equipment (PPE) and for hospitals, medical transport, emergency departments (EDs), and primary care settings had to be developed and disseminated on a just-in-time basis. Treatment guidelines also had to be developed, updated, and disseminated as knowledge grew.

***For population-based disease control***. *The authority and practical ability to adapt existing (or develop new, if necessary) policies and guidance to prevent the spread of communicable disease, including personal hygiene, quarantine, social distancing, and border controls*. During the Ebola outbreak, border screening policies and procedures regarding epidemiologic risks had to be developed, updated, and implemented to stay consistent with evolving knowledge. For example, during the H1N1 pandemic, policies and procedures regarding antiviral and vaccine priorities had to be developed and implemented. Advice about hand washing and other aspects of personal hygiene needed to prevent spread of the virus in the population also had to be developed and disseminated to the public.

***Enforcing laws and regulations****. The ability to enforce laws and regulations required for disease control and prevention including the IHR, EU regulations, and member states' laws and regulations.* Effective implementation of disease control policies, regardless of whether policies were in place before the event or were developed during the event, depends on the existence of appropriate laws and regulations, as well as procedures for modifying and implementing such policies. During the H1N1 pandemic, EU member states reviewed, but did not implement, social distancing policies. The volcanic ash event required airspace closures, relaxation of noise restrictions (so planes could be re-routed to different airports), and efforts to ensure passengers' rights. The melamine incident required import restrictions on contaminated products. During the Ebola crisis, there were numerous legal and administrative barriers to the international deployment of experts and teams, security, insurance, logistical support, medical evacuation, and international responders, which had to be addressed.

***3. Health Services***

This group of capabilities addresses prevention and treatment services delivered to individuals by the member states' health sector. This includes provision of vaccines and other countermeasures to the general public and to healthcare workers, as well as physical and mental health care for those affected by mass-casualty and long-running incidents. The medical countermeasures, supplies, and equipment needed to provide these services are considered capacities, but procuring additional supplies, managing the stockpile, and distributing them during a crisis are critical capabilities and thus are included in this section. The most effective and efficient means for delivering healthcare services to individuals—whether preventive or therapeutic—will depend on the nature of the threat and the way in which the public and private sector is organized, as the organization of the health sector varies markedly among member states. Thus, these capabilities focus on what must be accomplished during an event, rather than on how to accomplish it. The WHO Regional Office for Europe's toolkit for assessing health system capacity for crisis management^10^ provides more detail on how to assess and improve member states' preparedness in this regard.

***Preventive services***. *The ability to provide vaccines and other prophylactic measures to the population*. During an emergency, preventive measures must often be rapidly delivered to large numbers of at-risk individuals, requiring collaboration between the public and private sectors. For example, a challenge presented during the H1N1 outbreak was administering a newly developed pandemic vaccine to the public as soon as it became available, which occurred in autumn 2009. Member states accomplished this through a variety of approaches, including public health vaccination clinics, distribution through primary care providers, and other means as their health systems permitted. Routine prevention, such as childhood vaccination programs, are not part of this capability, but a member state's ability to conduct them effectively on a routine basis can be an indicator of its ability to provide prophylactic measures during an emergency.

***Medical surge***. *The ability to (1) evaluate and care for a markedly increased volume of patients and (2) manage patients requiring unusual or specialized medical evacuation or care.*^10^ Mass-casualty incidents, defined as events that generate more patients at one time than locally available resources can manage using routine procedures, require either high-volume or specialized physical and mental health treatment for those affected or, in the most severe cases, both. During the Ebola event, for instance, the challenge focused much more on specialized care: the evacuation, internal transport, and treatment of healthcare workers who were infected while working in West Africa. In some member states, this was accomplished primarily through public health sector Medevac and ambulance services. The number of Ebola patients treated in Europe was limited, and they were, for the most part, successfully treated at specially prepared hospitals. However, the event reminded hospital officials that treating more than one case per hospital would put an enormous strain on healthcare resources. This capability also includes the provision of temporary shelter to individuals evacuated from affected sites, as was necessary during the sludge and volcanic ash events.

***Management of medical countermeasures, supplies, and equipment****. The ability to procure, distribute, and manage countermeasures, supplies, and equipment, including PPE, during an incident.* Supplies and equipment per se are capacities, but member states' ability to use them effectively during a crisis is a response capability. For instance, while the number of Ebola cases in Europe was small, the outbreak highlighted existing challenges regarding limited availability of PPE, specialized ambulances, and other equipment.

***Care for healthcare workers and emergency responders****. The ability to provide preventive and medical services to address physical and mental health needs of healthcare workers and emergency responders.* Healthcare workers and emergency responders have special needs for preventive and medical care because of their exposure to pathogens and environmental contaminants. Moreover, because they put themselves at risk voluntarily and are necessary for maintaining continuity of operations, these workers may deserve priority when facing shortages. Priority for prophylaxis and treatment for responders and their families can also be important to ensure their response during a crisis. The 2009 H1N1 outbreak, for instance, highlighted the priority healthcare and emergency workers received for antivirals and pandemic vaccine when it became available. During the sludge incident, workers required gloves, protective dust masks, and footwear for use during the cleanup operation.

***4. Coordination and Communication***

This group of capabilities comprises efforts to communicate within the public health emergency preparedness system to coordinate and manage a complex system's response to a cross-border threat. The first 3 groups of capabilities describe *what* must be accomplished during a crisis; the capabilities are not ends in themselves, but rather describe *how* member states achieve these ends. Effective leadership and governance structures are important for crisis management, but because these factors vary across member states, here the focus lies on key aspects of coordination and communication needed during an emergency. Similarly, it is recognized that existing regulations and resource constraints may limit the amount of coordination, but these and other factors that explain why a member state was not able to coordinate their efforts are appropriate topics for a critical incident analysis.

***Crisis management****. Employing a systematic approach to organize and manage resources and responsibilities for addressing all aspects of emergencies, including continuity of operations.* Crisis management is a comprehensive category, referring to a member state's overall ability to balance the demand for immediate short-term actions with the maintenance of long-term operations. The Ebola event, for instance, provided a test of national response platforms and coordination efforts, essential for effective crisis management and the importance of leadership and governance. During the heat wave, in contrast, communication signals got lost, communiqué writing took days, and officials were slow to organize meetings and set up networks for making and acting on decisions. Scientific organizations struggled during the heat wave to modify their response from the usual methodology of carefully designed studies to rapid turnaround analyses, so as to inform the immediate response.

***Communication with healthcare providers****. Communication between public health agencies and healthcare providers, especially regarding surveillance protocols, prevention and treatment guidance, and other matters, to ensure coordination of prevention and treatment efforts.* During the Ebola outbreak, public health agencies communicated regularly with hospitals and healthcare providers regarding risk characterization, case definitions, infection control practices, and treatment protocols. During the H1N1 pandemic, Italian public health officials and primary care providers had different views about prevention strategies, complicating efforts to involve primary care providers in mass vaccination efforts.^19^ During the heat wave, an official instructed some hospitals to implement emergency measures outside of official channels, causing confusion.

***Communication with emergency management, public safety, and other sectors****. Communication between public health and other sectors to ensure coordination of prevention and treatment efforts*. During the H1N1 pandemic, public health agencies worked with other agencies to coordinate vaccine distribution points, delegate prescription, and other logistical matters. Member states also reported using just-in-time drills and simulations to prepare for Ebola cases.

***Communication with other public health agencies at the global, European, national, and subnational levels****. Communication between public health agencies at all levels to ensure coordination of prevention and treatment efforts*. This capability focuses on communication within member states, but effective response to cross-border threats requires communication and coordination at both the global and European levels. Member states must be able to participate in these discussions, translate and tailor the results to their own situations, and communicate them to relevant national and subnational parties. For example, during the *E. coli* outbreak, a European working group was established early in the response. ECDC issued rapid risk assessments and epidemiologic updates, with ECDC's Food and Water Borne Disease network playing an essential role. During the Ebola outbreak, the Health Security Committee (constituted according to Decision 2013/1082) consulted daily for joint coordination of EU response, exchange of best practices, and support to medical evacuation; in addition, an EU task force was established through the EU Commission's Emergency Response Coordination Centre to provide support in field operations in West Africa.

***5. Emergency Risk Communications***

The focus of this group of capabilities is the real-time exchange of information, advice, and opinions between experts or officials and people who face a threat (hazard) to their survival, health, or economic or social well-being. The ultimate purpose of this group of capabilities is to ensure everyone at risk is able to make informed decisions for mitigating the effects of the threat (hazard) and to take protective and preventive action.

***Address communication inequalities.*** *The ability to address differences across population groups regarding how the message is received, processed, and acted on, due to the diverse socioeconomic and cultural characteristics of the population affected by the emergency.*^20^ Communication inequalities are defined as differences in how segments of the population access, process, interpret, and react to information, specifically stemming from differences in individuals' demographic characteristics (eg, age and gender) and social factors (eg, socioeconomic positions). These differences may result in inequalities within populations regarding levels of risk perception and ability to acquire knowledge during a crisis, potentially affecting compliance with recommended behaviors.^21^ This capability addresses such inequalities, as well as cultural and societal barriers, in the cognitive processing of the information received during a crisis. It refers to the capability of the system to use the most appropriate content and trusted channels of communication across population groups and to identify strategies to overcome linguistic barriers when needed. While communication inequalities are context specific and need to be investigated during the response to each emergency, the literature shows some consistencies among factors such as socioeconomic position, race/ethnicity, language spoken at home, and place of residence in exerting an impact on people's ability to receive and use the information provided to them.^22,23^

***Use dynamic listening, gather evaluation data, and manage rumors.*** *The ability to disseminate messages that are clear and to collaborate with other organizations, including health professionals and local leaders, to disseminate the message using appropriate channels and messengers.* Communication is a 2-way process, consisting of both announcements to the public and monitoring information released by the media, shared on social media and other outlets, and how such information is processed and understood by the public. “Dynamic listening” refers to processes related to understanding the public's concerns and reactions, which is necessary for disseminating messages that address these concerns. For instance, during the 2009 H1N1 pandemic, member states realized that the public was concerned about insufficient stockpiles of antivirals and vaccines, leading public officials to reassure the public that the necessary supplies were available.

***Communicate risk in an accurate, transparent, and timely manner****. The ability to provide information to the public in a timely manner, taking into account the measurement of actual risk, uncertainty, and populations' risk perceptions.* There is often a tradeoff between accuracy and timeliness: Officials often want to respond to public concerns quickly, before all facts are known. This happens especially in situations in which public concern is high and risk is low, such as in the United States and Europe during the Ebola outbreak. However, communicating too quickly can subsequently create confusion in the population if the original message must be adapted as the facts develop,^24,25^ as happened during the 2011 hemolytic uremic syndrome outbreak in Germany.^26^ This capability includes the ability to acknowledge uncertainty when needed and avoid delays in the release of information.^27^ Timeliness has been related to government's transparency with regard to avian flu as well as the H1N1 pandemic.

***Foster and maintain trust.*** *The ability to deliver messages that foster citizens' trust in the way the government officials handle the emergency.* Levels of trust in the government have been associated with compliance with recommended behaviors, as noted in the Netherlands during the H1N1 pandemic. This capability^28^ includes the ability to identify sources of information trusted by the public^29^ (ie, communication channels, public officials, specific individuals, and community leaders) as well as the ability to educate the public on the roles and responsibilities of various organizations involved in the response.

## Discussion

The ECDC logic model presented in this article, with its associated capabilities and capacities, represents a theoretical framework for identifying *what* to measure in capacity inventories, exercises, critical incident analyses, and other approaches to assessing public health emergency preparedness, not *how* to measure them. Focusing on a common set of capacities and capabilities for measurement allows for comparisons both over time and between member states, which is important for enhancing learning and sharing results across all approaches and, ultimately, for identifying both strengths and areas for improvement in public health emergency preparedness in the EU. The capabilities in the logic model can be useful for structuring a critical incident analysis that covers all relevant dimensions of preparedness.

This article represents the first step in ECDC's plans to eventually develop and deliver competency-based training programs to assist member states in fulfilling their responsibilities under Decision 1082. As training budgets, time, and personnel backfill resources are all limited, public health officials need an efficient and highly practical educational approach that effectively addresses core readiness competencies. However, competencies are characteristics of individuals who work in the public health emergency preparedness system, whereas capacities and capabilities are system-level characteristics. To bridge this gap, our subsequent work will review the strengths and weaknesses of approaches for assessing countries' capabilities and capacities, as specified in the ECDC logic model, such as drills, simulation exercises, and critical incident analyses. We will then develop detailed competencies and related knowledge, skills, and abilities for specific staff roles and capabilities. In this way, EU member states will be able to identify training needs and ECDC can deliver training where it is most needed.
